# Hypoxia inducible factor-1 mediates expression of miR-322: potential role in proliferation and migration of pulmonary arterial smooth muscle cells

**DOI:** 10.1038/srep12098

**Published:** 2015-07-13

**Authors:** Yan Zeng, Hongtao Liu, Kang Kang, Zhiwei Wang, Gang Hui, Xiaoying Zhang, Jiasheng Zhong, Wenda Peng, Ramaswamy Ramchandran, J. Usha Raj, Deming Gou

**Affiliations:** 1 Shenzhen Key Laboratory of Microbial Genetic Engineering, Shenzhen Key Laboratory of Marine Bioresourse and Eco-environmental Science, College of Life Sciences, Shenzhen University, Shenzhen, Guangdong, 518060, China; 2 Key Laboratory of Optoelectronic Devices and Systems of Ministry of Education and Guangdong Province, College of Optoelectronic Engineering, Shenzhen University, Shenzhen, Guangdong, 518060, China; 3 Department of Cardiovascular Surgery, Shenzhen Sun Yat-Sen Cardiovascular Hospital, Shenzhen, Guangdong, 518000, China; 4 Department of Chest Surgery, Peking University Shenzhen Hospital, Shenzhen, Guangdong, 518000, China; 5 Department of Pediatrics, University of Illinois at Chicago, Chicago, IL 60612, U.S.A

## Abstract

There is growing evidence that microRNAs play important roles in cellular responses to hypoxia and in pulmonary hypertensive vascular remodeling, but the exact molecular mechanisms involved are not fully elucidated. In this study, we identified miR-322 as one of the microRNAs induced in lungs of chronically hypoxic mice and rats. The expression of miR-322 was also upregulated in primary cultured rat pulmonary arterial smooth muscle cells (PASMC) in response to hypoxia. We demonstrated that HIF-1α, but not HIF-2α, transcriptionally upregulates the expression of miR-322 in hypoxia. Furthermore, miR-322 facilitated the accumulation of HIF-1α in the nucleus and promoted hypoxia-induced cell proliferation and migration. Direct targeting BMPR1a and smad5 by miR-322 was demonstrated in PASMCs suggesting that downregulation of BMP**-S**mad signaling pathway may be mediating the hypoxia-induced PASMC proliferation and migration. Our study implicates miR-322 in the hypoxic proliferative response of PASMCs suggesting that it may be playing a role in pulmonary vascular remodeling associated with pulmonary hypertension.

Pulmonary arterial hypertension (PAH) is a progressive and often life-threatening disease characterized by elevation of pulmonary arterial pressure and pulmonary vascular resistance and vascular remodeling involving the dysregulation of various components of the vessel wall^1^,^2^,^3^. The smooth muscle layer of the vessel wall plays a prominent role in the pathogenesis of PAH with extension of smooth muscle into smaller, non-muscular pulmonary arteries within the respiratory acinus, a common feature of all forms of PAH-associated remodeling^1^. Pulmonary arterial smooth muscle cells (PASMC) proliferate and migrate with medial wall thickening, resulting in decreased luminal diameter and ultimately obstruction of resistance pulmonary arteries^1^,^4^. Chronic hypoxia is a well-known stimulus for abnormal proliferation and migration of vascular smooth muscle cells and vascular remodeling in patients with PAH^5^,^6^,^7^. Although the cellular and molecular mechanisms involved in these proliferative and migratory responses are still not completely understood, there is very strong evidence that hypoxia-inducible transcription factors (HIF) are critically involved^8^,^9^.

Activation of hypoxia-inducible factor 1α (HIF-1α) is the primary hypoxia-driven signaling pathway in the pulmonary vasculature^10^,^11^. HIF-1α is a heterodimeric transcription factor that is composed of a regulatory α subunit and a constitutive β subunit (HIF-1β/ARNT). HIF-1α is selectively stabilized under hypoxia, so it can translocate into the nucleus to combine with the β subunit and bind to the hypoxia responsive elements (HREs) and activate transcription of genes that promote vascular cell growth and development, glycolytic metabolism and cell cycle events^12^. In heterozygous HIF-1α knockout mice, hypoxia-induced pulmonary hypertension and vascular remodeling are notably reduced^13^. Also, HIF-2α heterozygous deficient mice do not develop pulmonary hypertension even after exposure to prolonged hypoxia^14^. However, the key molecular and cellular pathways that are influenced by HIF are still being described.

MiRNAs are single-stranded, non-coding RNA molecules of about 20–26 nucleotides in length that regulate 50–60% of mammalian gene expression by interacting with the 3′-untranslated regions (3′-UTR) of specific mRNA targets and inhibiting translation^15^. MiRNAs play significant roles in the regulation of various cellular processes, including proliferation, migration, differentiation, and apoptosis^7^,^16^,^17^. Recently, miRNAs have been implicated in the development and progression of PAH, especially in the presence of hypoxic stress. Increased expression of miR-451 and miR-30c as well as differential expression of miR-21 and let-7a has been reported in rat models of PAH induced by monocrotaline and chronic hypoxia^18^. A recent study reported that miR-20a and miR-17, through the STAT3 pathway, participate in the regulation of morphogenetic protein receptor type 2 (BMPR2), which is a key determinant of idiopathic familial pulmonary hypertension^19^. Another study revealed that miR-20a inhibition could restore functional BMPR2 signaling and reduce vascular remodeling in hypoxia-treated mice^20^.

In this study, we investigated the role of miR-322 in hypoxia-induced cellular responses in rat PASMCs. We provide evidence that HIF-1α, but not HIF-2α, upregulates the transcription of miR-322 and that miR-322 may modulate proliferation and migration of PASMCs via the BMP-Smad pathway.

## Results

### Hypoxia upregulates expression of miR-322 in lungs and PASMCs

**Figure 1:**
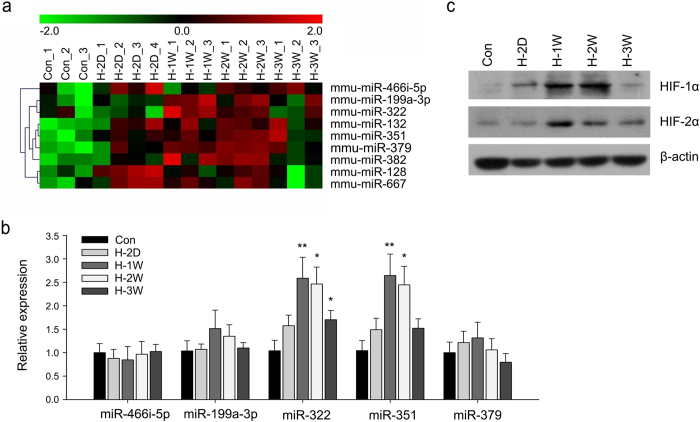
miR-322 is induced by hypoxia in mouse lung. (**a**) Differential expression of 1040 miRNAs in lung tissue from mice exposed to normoxia (21% O_2_)(Con) or hypoxia (10% O_2_) for 2 days (H-2D), 1 week (H-1W), 2 weeks (H-2W) and 3 weeks (H-3W) (n = 3 or 4 for each group). Cluster analysis of miRNAs expression from individual specimens were assessed by microarray analysis. (*p* < 0.05 compared with normoxic controls). (**b**) Validation of miRNAs upregulated by hypoxia by real-time PCR assay. Bar charts showing the relative expression level by normalizing to the respective normoxia control (Con). Data are shown as means ± SD, *p < 0.05, **p < 0.01. (**c**) Hypoxia results in stabilization of HIF-1α and HIF-2α proteins in the lung. Representative immunoblots showing the lung HIF-1α and HIF-2α levels in normoxia and hypoxia-exposed mice. β-actin levels served as loading control. The full-length blots with these antibodies were presented in supplementary Figure S1. All gels have been run simultaneously under the same experimental conditions.

To determine the lung miRNA profile in chronic hypoxia (10% O_2_)-induced PH in mice, we performed a microarray analysis. The microarray profile revealed that several miRNAs, including miR-466i-5p, −199a-3p, −322, −351 and −379, were significantly upregulated after 3-weeks of hypoxic exposure (Fig. 1a). Then, an independent quantitative real-time PCR (qRT-PCR) assay was carried out to confirm the expression pattern of these miRNAs, normalized to sno202. The results showed that the expression of miR-322 and miR-351 was increased significantly with the duration of hypoxia exposure (Fig. 1b). Western blot analysis showed increased expression of HIF-1α and HIF-2α in lung tissue extracts in response to hypoxia, with β-actin serving as an internal control (Fig. 1c).

**Figure 2:**
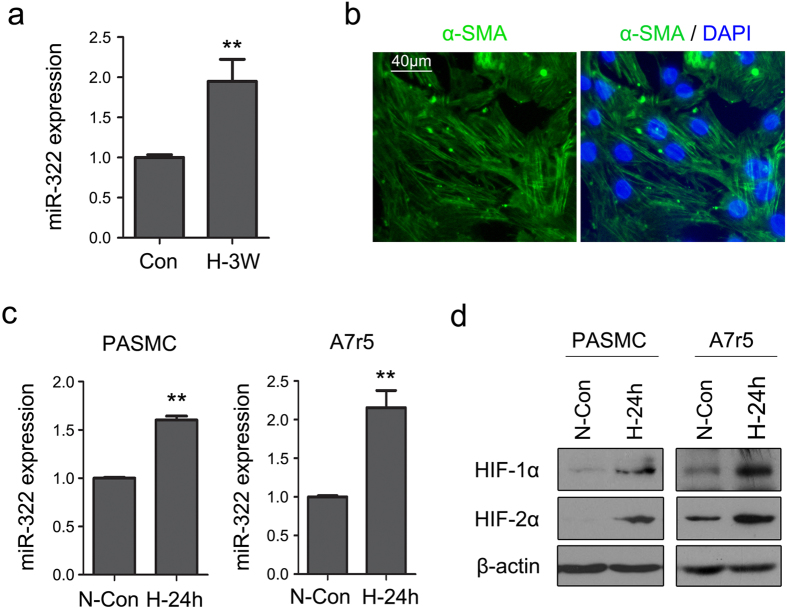
Hypoxia upregulates expression of miR-322 in rat PASMC and A7r5 cells. (**a**) Quantitative RT-PCR assay of miR-322 in rat lungs exposure to hypoxia (n = 3 for each group). Data were shown as mean ± SD, **p < 0.01 compared to Con. (**b**) Purity of rat PASMCs in primary culture. Smooth muscle cells were isolated from rat pulmonary artery and immunostained with smooth muscle α-actin (α-SMA, green) antibody and DAPI (blue) to assess purity. Scale bar, 40 μm. (**c**) Rat PASMCs and A7r5 cells were exposed to normoxia (21% O_2_) (N-Con) or hypoxia (3% O_2_) for 24 h (H-24 h). The relative levels of miR-322 was estimated by real-time PCR. Data are shown as means ± SD relative to respective normoxia controls, **p < 0.01. (**d**) Representative western blot showing HIF-1α, HIF-2α and β-actin (as loading control) protein levels in rat PASMCs and A7r5 cells exposed to either normoxia or hypoxia for 24 h. The full-length blots with these antibodies were presented in supplementary Figure S2. All gels have been run simultaneously under the same experimental conditions.

Next we determined whether hypoxia-induced expression of miR-322 in rat lung and *in vitro* cultured PASMCs parallels the mouse lung miRNA profile. As shown in Fig. 2a, miR-322 level in rat lungs was increased about 2-fold after 3-weeks hypoxic treatment. PASMCs isolated from Sprague-Dawley rats were determined to be 98% pure by staining with smooth muscle-specific α-actin (α-SMA) (Fig. 2b). PASMCs were grown either in normoxia (21% O_2_) or hypoxia (3% O_2_) for 24 h, and miRNA levels were measured by qRT-PCR. As shown in Fig. 2c, the expression of miR-322 was upregulated 1.6-fold in hypoxia compared to normoxia. A similar study was carried out in A7r5 cells a smooth muscle cell line derived from rat thoracic aorta. In these cells miR-322 expression was increased about 2.2-fold in hypoxia. The increase in HIF-1α and HIF-2α levels in hypoxia was also confirmed in PASMCs and A7r5 cells (Fig. 2d).

### miR-322 is transcriptionally regulated by HIF-1α

**Figure 3:**
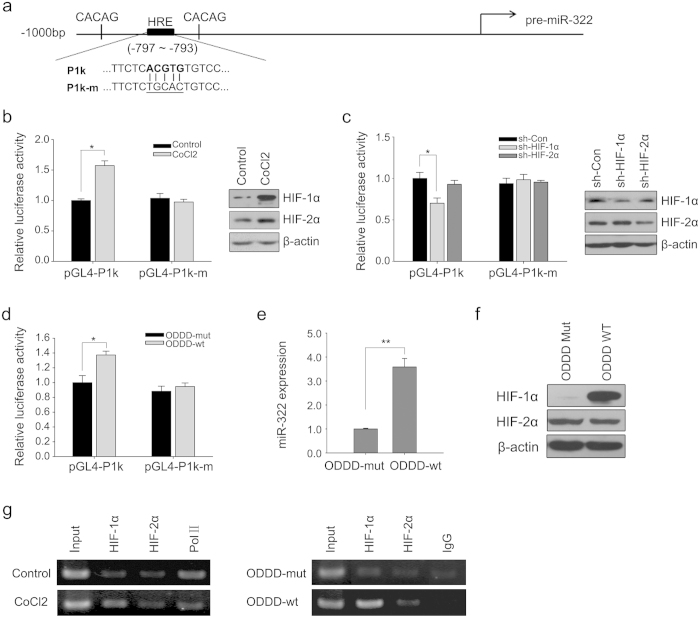
Hypoxia induces miR-322 promoter activity in a HIF-1α-dependent manner. (**a**) Schematic diagram of cloned rat putative promoter region of miR-322, stretching from upstream 1000 bp. The position of the putative HRE matching the core sequence (A/G)CGTG is labeled in box, between two functional CACAG elements (P1k). A mutant promoter was constructed with the sequence as shown below (P1k-m). (**b**) Luciferase reporter assays of miR-322 promoter activity with both wild (pGL4-P1k) and mutant constructs (pGl4-P1k-m) in the presence or absence of CoCl_2_ (200μM) (left panel); Under these conditions, the protein levels of HIF-1α and HIF-2α were stabilized after CoCl_2_ treatment in the cells as determined by western blot analysis and normalized to β-actin levels (right panel). (**c**) miR-322 promoter activity assessed by luciferase reporter assay after CoCl_2_ treatment was diminished by HIF-1α knockdown. Cells were transduced with shRNA targeting HIF-1α (shHIF-1α) or HIF-2α (shHIF-2α) and miR-322 promoter activity was determined in the luciferase reporter assay after transfection of wild type and mutant constructs and CoCl_2_ treatment (left panel) as described under Methods; Control (sh-Con) or HIF-1α/-2α shRNA-transfected cells were harvested for protein analysis by western blot to determine knockdown specificity (right panel); *p < 0.05 compared with sh-Con. (d & e) A7r5 cells were transfected with recombinant adenoviruses expressing oxygen-dependent degradation domains (ODDD-wt) or mutated ODDD (ODDD-mut) under normoxic conditions. The influence on the miR-322 promoter reporter activity (**d**) and the endogenous expression levels (**e**) of miR-322 were determined by real-time PCR. All the bar plots represent means ± SD. *p < 0.05, **p < 0.01,compared with ODDD-mut. (**f**) Western blot for HIF-1α and HIF-2α in ODDD–transfected A7r5 cells after 24 hours. β-actin served as loading control. The full-length blots with these antibodies were presented in supplementary Figure S3. All gels have been run simultaneously under the same experimental conditions. (**g**) HIF-1α dynamic binding on the HRE site (−797 to −793) on the miR-322 promoter. ChIP assays were performed with indicated antibodies in the absence or presence of CoCl_2_ (left), or transfected with ODDD-wt or ODDD-mut (right) as described under Methods. Representative gel with input lanes showing products after PCR amplification and before immunoprecipitation.

Since HIF-1 and -2 mediate most of the cellular responses to hypoxia, we hypothesized that the hypoxia-induced increase in expression of miR-322 is HIF-related. We searched for hypoxia-responsive elements (HRE) in the putative promoter sequence, 1000 bp upstream from the rat pre-miR-322. One potential HIF-binding site was identified within this region, containing a HRE core sequence (A/G)CGTG stretching from −797 bp to −793 bp. Two functional HRE elements (CACAG) are located 138 bp upstream and 55 bp downstream, respectively (Fig. 3a). We constructed both wild and HRE-mutant promoter-driven luciferase reporter plasmids (pGL4-P1k and pGL4-P1km, respectively) and transfected A7r5 cells to determine whether HIF-1/2α influences the miR-322 promoter activity. Cells were treated with cobalt chloride (CoCl_2,_ 200 μM, 24 h), a hypoxia-mimetic compound that has been shown to stabilize HIF protein in PASMC^21^. The results show that the native promoter but not the mutant promoter of miR-322 was activated by CoCl_2_ (Fig. 3b). Moreover, CoCl_2_-induced expression of HIF-1α and -2α in A7r5 cells was confirmed by western blotting (Fig. 3b). We further tested the roles of HIF-1α and -2α in regulation of miR-322 promoter activity using a gene knockdown approach. As shown in Fig. 3c, shRNA targeting of HIF-1α almost completely abolished the activation of miR-322 promoter reporter induced by CoCl_2_, but HIF-2α silencing had no effect. And both shRNAs had no effect on the mutant promoter. Western blot analysis confirmed decreased expression of HIF-1α or HIF-2α after CoCl_2_-treatment with specific shRNA (Fig. 3c). This indicates that the HRE site within the miR-322 promoter is required for HIF-1α-induced upregulation in hypoxia.

To further assess whether HIF-1α affects the transcription of miR-322, we transfected A7r5 cells with an adenoviral vector expressing modified oxygen dependent degradation domains (ODDD-wt) that express a HIF-1α ODDD domain from amino acids 531 to 575. This domain competes with endogenous HIF-1α/-2α for pVHL recognition and degradation^22^. Another adenoviral vector expressing mutant ODDD (ODDD-mut), with a mutation of proline 564 to alanine, was constructed which can be recognized by the VHL complex for HIF-2α but not HIF-1α^22^. This was used as a negative control for HIF-1α stabilization. Luciferase assays showed that HIF-1α stabilization specifically activated miR-322 promoter activity but not that of the mutant (Fig. 3d). As shown in Fig. 3e,f, qRT–PCR and western blotting revealed that the endogenous expression levels of miR-322 also dramatically increased with HIF-1α accumulation.

Using chromatin immunoprecipitation (ChIP) assay, we determined the binding of HIF-1α to the miR-322 promoter with and without CoCl_2_ treatment or with ODDD-wt/-mut overexpression. As shown in Fig. 3g, binding of HIF-1α to the HRE site increased in A7r5 cells, but HIF-2α binding did not exhibit any change. We validated the system by using antibodies against IgG or RNA polymerase II (Pol II) and primers for a region lacking HRE site (data not shown). The ChIP assays indicated that HIF-1α directly binds to the HRE site on the miR-322 promoter *in vitro*. Altogether, the results above demonstrated that miR-322 is transcriptionally regulated by the hypoxia-responsive factor HIF-1α in hypoxia.

### miR-322 facilitates stabilization of HIF-1α

**Figure 4:**
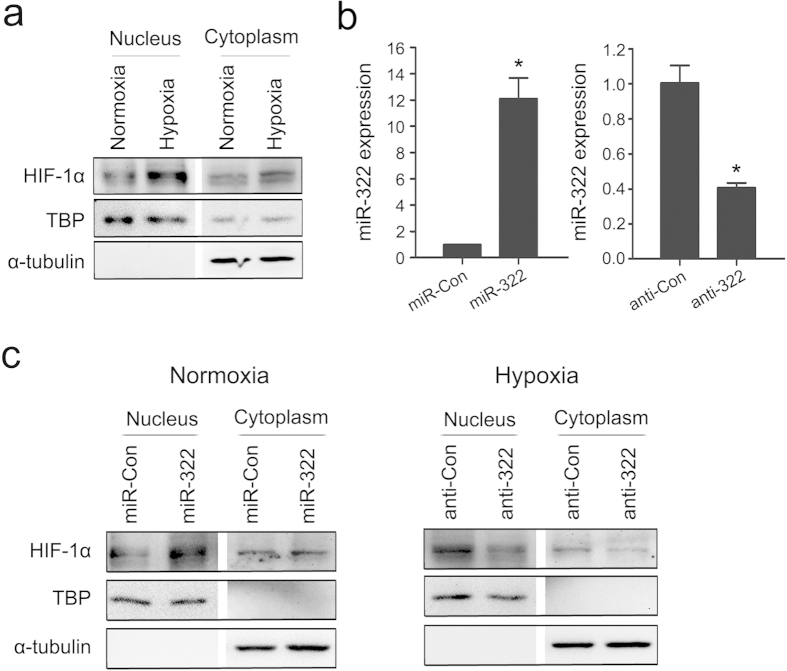
miR-322 stimulates the accumulation of HIF-1α. (**a**) Western blotting analysis of HIF-1α subcellular expression in rat PASMCs exposed to hypoxia for 24h. TBP (TATA box Binding Protein) and α-tubulin were used as internal controls for the nuclear and cytoplasmic fractions, respectively. (**b**) Quantitative real-time PCR quantification of miR-322 in stably transfected rat PASMCs. Cells were transfected with recombinant lentiviral particles expressing miR-322 (miR-322) or control lentiviral vector (miR-Con), or with lentivirus of dTud construct against mature miR-322 (anti-322) or scramble control vector (anti-Con). Data are shown as means ± SD relative to respective controls, *p < 0.05 compared with controls. (**c**) Left: Immunoblotting to determine HIF-1α expression in cells transfected with lentivirus expressing miR-322 or control lentivirus under normoxia. Right: Immunoblotting to determine HIF-1α expression in PASMCs transfected with anti-322 construct or scramble control and exposed to hypoxia. The full-length blots with these antibodies were presented in supplementary Figure S4. All gels have been run simultaneously under the same experimental conditions.

Ghosh *et al.* have reported that human miR-424, the homolog of rodent miR-322, stabilizes HIF-1α leading to its accumulation in the nucleus in human umbilical vein endothelial cells (HUVEC)^23^. Therefore, we determined whether manipulating miR-322 expression could affect the levels of HIF-1α in rat PASMCs. Cells were transfected with recombinant lentivirus expressing miR-322 (miR-322), or no miRNA sequence as a negative control (miR-Con). Stably transfected cells were generated by puromycin selection. As shown in Fig. 4a, exposure to hypoxia induced HIF-1α accumulation in the nucleus of PASMCs. qRT-PCR revealed an over 12-fold increase in miR-322 in the cells overexpressing miR-322 compared with control cells (Fig. 4b). Western blot showed that the accumulation of HIF-1α increased in the nucleus when overexpressing miR-322 in normoxia (Fig. 4c). Cells were also transfected with dTud constructs for miR-322 knockdown studies. As shown in Fig. 4b, cells with miR-322 inhibition (anti-322) exhibited significantly reduced expression in miR-322 compared to its control cells (anti-Con). Knockdown of miR-322 abolished the stabilization of HIF-1α in PASMCs under hypoxic condition (Fig. 4c). Taken together these results indicate that hypoxia-induced miR-322 is involved in regulating the stability of HIF-1α in PASMCs.

### miR-322 promotes hypoxia-induced proliferation and migration in PASMCs

**Figure 5:**
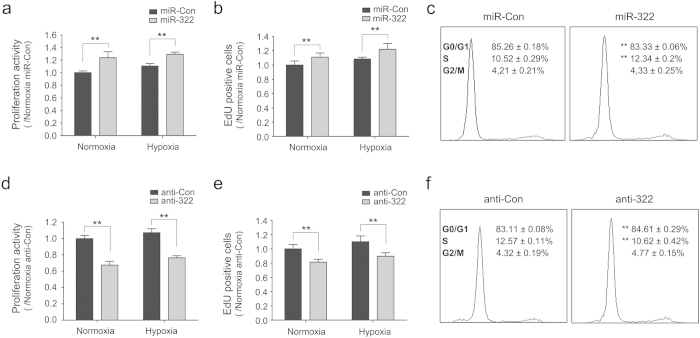
miR-322 promotes proliferation of PASMCs. (**a, d**) PASMCs were transduced with miR-Con, miR-322 or anti-Con, anti-322 and exposed to hypoxia (3% O_2_) or normoxia for 24h before measuring proliferative index as described under methods. Bar chart represents fold change in OD 490nm readings relative to miR-Con or anti-Con in both normoxic and hypoxic conditions.** p < 0.01 compared to miR-Con or anti-Con. (**b, e**) EdU incorporation assay showing proliferation activity of miR-322-overexpressed or inhibited cells and each control cells under normoxia and hypoxia. Data are shown as means ± SD, **p < 0.01 compared to miR-Con or anti-Con. (**c, f**) Staining of transfected cells with propidium iodide (PI) to assess cell cycle phase distribution by DNA content as described under Methods. Data are shown as means ± SD, **p < 0.01 compared to miR-Con or anti-Con.

To understand the consequences of increased miR-322 in hypoxia, we investigated the effects of miR-322 overexpression and knockdown on the proliferation and migration in rat PASMCs. As shown in Fig. 5a, stable overexpression of miR-322 significantly accelerated the proliferation rate of PASMCs compared to its control cells both under normoxia and hypoxia by MTS assay. EdU incorporation assay, a specific assay that labels replicating cells, also showed higher fraction of proliferating cells in miR-322-expressing cells compared with control cells (Fig. 5b). FACS analysis of PI-stained cells indicated that the proportion of cells in S and G2/M phase was significantly increased, and cells in G1 phase decreased by a comparable degree (Fig. 5c). To further confirm these results, we performed the proliferation assay in PASMCs after miRNA knockdown. In contrast with miRNA overexpression studies, proliferation was significantly reduced in anti-322 transfected cells compared to that of control cells, both when exposed to normoxia or hypoxia (Fig. 5d,e,f). These data indicate that miR-322 can significantly accelerate the proliferation of PASMCs.

**Figure 6:**
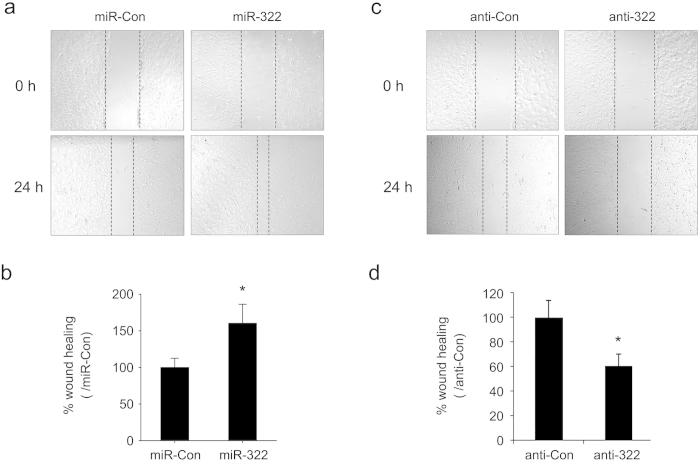
miR-322 promotes migration of PASMCs. (**a, c**) Representative images of wound-healing assay showing migration of rat PASMC either with miR-322 overexpression or knockdown. (**b, d**) Bar chart showing relative decreased wound width after 24 h. Data are shown as mean ± SD. *p < 0.05 compared to miR-Con or anti-Con.

Cell migration was assessed through a wound-healing assay. As shown in Fig. 6, the decrease in the width of the scratched wound was larger (~ 60%) in miR-322 transfected cells than in control cells after 24 h (Fig. 6a,b), and knockdown of miR-322 led to a slower decrease in wound healing compared with anti-Con transfection (~ 40%) after 24 h (Fig. 6c,d). Taken together these data demonstrate that hypoxia-induced miR-322 can promote migration of PASMCs.

### miR-322 participates in hypoxia-mediated BMP signaling

**Figure 7:**
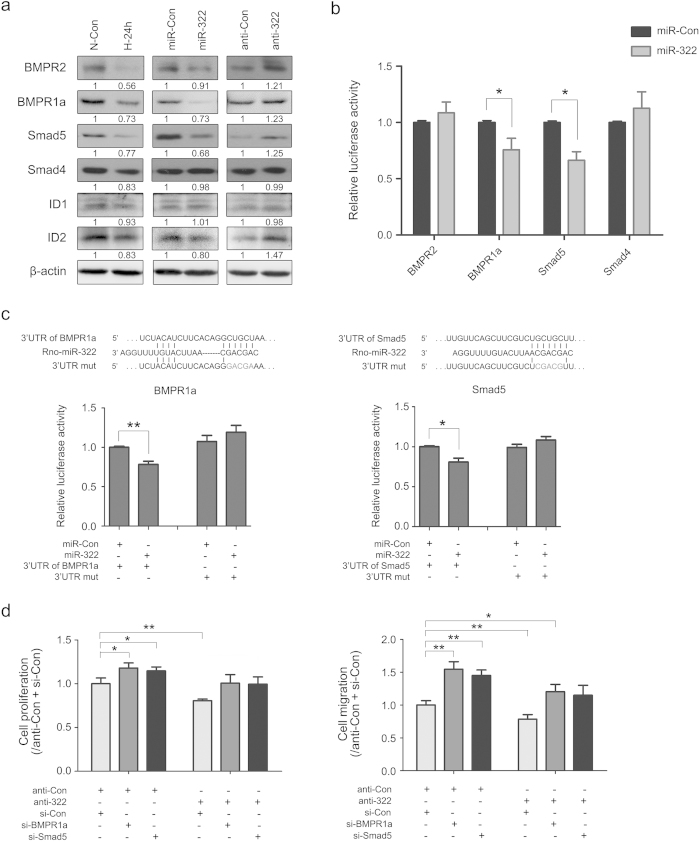
miR-322 regulates BMP mediated signaling. (**a**) Expression of BMP signaling related components in rat PASMCs. Left: representative western blot for hypoxia-dependent decrease of BMP signaling factors; Middle and right: Overexpression/ knockdown of miR-322 altered the protein level of factors in BMP signaling. The full-length blots with these antibodies were presented in supplementary Figure S5. All gels have been run simultaneously under the same experimental conditions. (**b**) Reporter gene studies on the interaction between miR-322 and 3′-UTR of the predicted targets in 293A cells. Luciferase activities were measured at 48 h after cotransfecting with the 3′-UTR constructs and the miR-322 overexpressing vectors or its vector control. (**c**) Validation of BMPR1a and Smad5 as direct targets of miR-322. Upper panel: conserved miR-322 binding sites in the 3′-UTR of Smad5 and BMPR1a along with the mutation site; Bottom panel: 3′-UTR luciferase reporter assay with targets and their mutant along with miR-322/miR-Con overexpressing vectors. Bar charts of luciferase reporter analysis represent means ± SD (n = 3), *p < 0.05, **p < 0.01 compared with miR-Con/3′UTR of targets. (**d**) Rescue experiments on EdU incorporation assay and wound-healing assay. Cells are transfected with miRNA inhibitor or/and siRNAs against BMPR1a or Smad5. Data are shown as means ± SD. *p < 0.05, **p < 0.01 compared to anti-Con + si-Con.

Dysregulated BMP signaling in PASMC and pulmonary artery endothelial cells (PAECs) is thought to have a pro-proliferative and pro-migratory effect through the Smad-dependent signaling pathway, a mechanism that is involved in hypoxia-induced PAH^24^,^25^. Therefore, we measured the expression of specific components of the BMP signaling pathway in rat PASMCs. Western blot analysis showed that the expression of BMPR2, BMPR1a (BMP type IA receptor), Smad4, Smad5, ID1 and ID2 (Inhibitors of DNA binding) in PASMCs was decreased in response to hypoxia (Fig. 7a, left panel). We also measured protein levels of these molecules involved in BMP signaling after overexpressing or silencing miR-322. Notably, the protein levels of BMPR1a, BMPR2, Smad5 and ID2 were decreased in miR-322 overexpressing cells under normoxia (Fig. 7a, middle panel). And they were all increased by miR-322 knockdown (Fig. 7a, right panel). These results suggest that hypoxia-induced miR-322 may play a regulatory role in PASMC proliferation and migration via the BMP signaling pathway.

To understand if the effect of miR-322 on BMP signaling is direct, we searched for potential binding sites on the 3′-UTR of the above proteins. Using an online tool (TargetScan, www.targetscan.org), putative seed match of miR-322 was predicted within the 3′- UTR of BMPR-2, BMPR1a, Smad5, and Smad4. Luciferase reporter assays with transfected 3′-UTR sequences along with miR-322 or miR-Con overexpressing vectors showed that only BMPR1a and Smad5 were repressed by miR-322 overexpression (Fig. 7b). Additional 3′-UTR mutation assays validated that miR-322 can directly target the predicted binding sites on the 3′-UTR of BMPR1a and Smad5 (Fig. 7c). To confirm that the regulation of miR-322 on cell proliferation and migration is achieved by targeting BMPR1a and Smad5, we carried out the following rescue experiments. As shown in Fig. 7d, the proliferation and migration of rat PASMCs, which were blocked by miR-322 inhibition (anti-322 and si-Con), were significantly increased by either BMPR1a or Smad5 knockdown (si-BMPR1a or si-Smad5 together with anti-Con). While, together transfection with si-BMPR1a or si-Smad5 almost completely rescued the proliferative and migratory repression caused by miR-322 inhibition. Taken together these results confirm that the positive role of miR-322 on PASMCs proliferation and migration could be fulfiled by post-transcriptionally regulating BMPR1a and Smad5.

## Discussion

**Figure 8:**
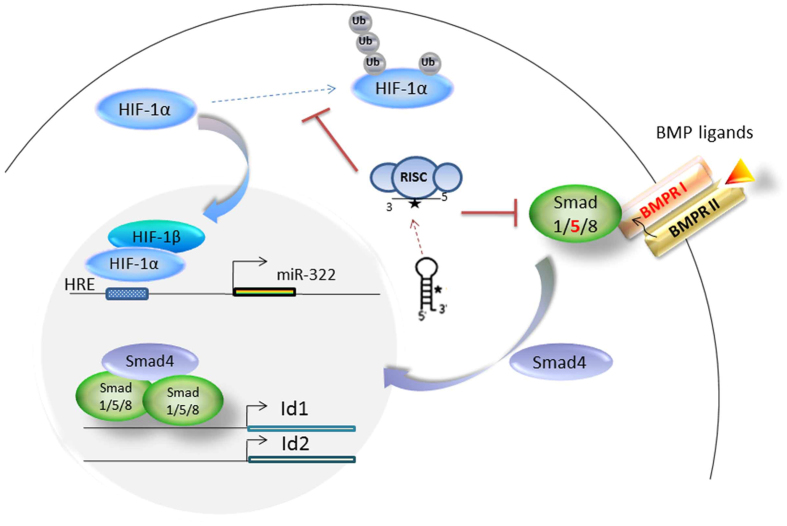
Model depicting the regulation of miR-322 in PASMCs response to hypoxia. Hypoxia-sensing via HIF-1 upregulates the transcription of miR-322, which then exerts positive feedback by facilitating stabilization of HIF-1α by targeting the molecules involved in its degradation for ubiquitination and degradation^23^. Dysregulation of BMP signaling by increased miR-322 promotes the proliferation and migration of PASMCs.

In this study, we have elucidated a possible mechanism by which miR-322 is upregulated in PASMCs during hypoxia. We have also characterized the biological effects and regulation of miR-322 in rat PASMCs (Fig. 8).

Hypoxia regulates the expression of a specific set of miRNAs previously termed hypoxamirs. Some hypoxamirs, such as miR-210, have a similar expression pattern in ubiquitous types of cells^23^, whereas others act as hypoxamirs only in specific types of tissues and cells. Caruso *et al.* have identified a number of hypoxamirs in rat lungs that are also affected by monocrotaline (MCT) treatment. Among these, miR-322 was found to be significantly upregulated during the course of development of PH^18^. Ghosh *et al.* have demonstrated that the human homolog of miR-322, has-miR-424, specifically increased in endothelial and vascular smooth muscle cells, but not in tumor cell lines, in response to hypoxia^23^. Here, we confirmed the hypoxia-induced increase in expression of miR-322 in rat PASMCs, highlighting miR-322 as the functional hypoxamirs in PASMCs.

A majority of adaptive changes to hypoxia at the transcriptional level are regulated by master transcription factors, HIF-1α and HIF-2α, in the pulmonary vasculature. These two hypoxia-induced factors are regulated by a similar mechanism that involves proline hydroxylation followed by ubiquitination through the VCBCR complex leading to proteasomal degradation^26^. It was reported that human miR-424, in HUVEC, promotes stabilization of HIF-α isoforms by targeting CUL2, which is a key scaffolding protein for assembly of the ubiquitin ligase system^23^. In this study, we found that miR-322 can also stimulate the accumulation of HIF-1α in PASMCs. More importantly, we found that HIF-1α upregulates the transcription of miR-322 by directly binding to the HRE site on its promoter. This indicates a positive feedback loop regulation between HIF-1α and miR-322 in pulmonary arterial smooth muscle cell, as shown in Fig. 8. However, HIF-2α seems not to be participating in this network. The various mechanisms involved in this pathway still need further investigation.

HIF-mediated hypoxic response in PASMC is associated with increased proliferation, migration and decreased apoptosis. Acutely, such adaptive responses to hypoxia are beneficial in preserving normal cellular function. However, long-term hypoxia ultimately leads to induction of molecular events that result in pulmonary arterial remodeling and increased pulmonary arterial pressures^27^. Proliferation and migration of PASMC is an essential feature of vascular remodeling in hypoxia-induced pulmonary hypertension. Our data show that miR-322 promotes the proliferation and migration of rat PASMCs both under normoxia and hypoxia. Furthermore, Ghosh *et al.*^23^ and Chamorro-Jorganes *et al.*^28^ have demonstrated that human miR-424 significantly increases migration and proliferation of endothelial cells and plays a positive role in post-ischemic vascular remodeling and angiogenesis. However, a recent study by Kim *et al.* reported in studies using pulmonary arterial endothelial cells from patients of idiopathic PAH and heritable PAH that endothelial APLN-mediated regulation of miR-424 that may have an anti-angiogenic function. Further studies are still needed to provide greater insights into the function and mechanism of this critical miRNA.

Dysregulated BMP/Smad signaling plays an important role in PAH. Mutations in BMPR2 are known to be present in a high proportion of patients with heritable PAH^29^,^30^,^31^. Recent observations also suggest that BMPR2 and BMPR1a expression is reduced in lungs of patients with idiopathic PAH, as well as in animal models of PH induced by MCT or chronic hypoxia^32^,^33^. Moreover, dysfunctional Smad1/5 signaling is present in PASMC isolated from patients with BMPR2 mutations and even in those without identifiable mutations^34^,^35^, and the resulting decrease in expression of ID1 and ID2 is believed to be a cause of proliferation and migration of PASMC^20^,^36^. BMP signaling is initiated by BMP ligand binding to BMPR1a and activating it and by the constitutively active BMPR2, with subsequent phosphorylation of the downstream signaling intermediaries Smad1 and 5^37^. The receptor-activated Smads then form a complex with their common partner Smad4 to enter the nucleus and modulate the transcription of target genes^38^. Our results show that in rat PASMCs all the BMP signaling factors are downregulated in response to hypoxia and miR-322 can affect the proliferation and migration of PASMCs by directly targeting BMPR1a and Smad5. In addition, the reduction or increase of other BMP-associated proteins caused by either overexpression or inhibition of miR-322 is perhaps achieved through an indirect pathway. All these data emphasize that miR-322 may affect the BMP signaling pathway to exert their pro-proliferation and pro-migratory activities in PASMCs.

## Materials and Methods

### Mice

All mice and rats were cared for in accordance with the University of Illinois at Chicago Animal Care Policy following the *Guide for the Care and Use of Laboratory Animals*. All animal experimental protocols were reviewed and approved by the Institutional Animal Care and Use Committee at the University of Illinois at Chicago. Chronic hypoxia exposure: Two-month-old adult C57/BL6 mice and Sprague-Dawley rats were exposed to normobaric hypoxia for different time intervals (2, 7, 14, and 21 days) in a plexiglass chamber provided with an oxygen controller by infusing nitrogen gas to maintain 10% oxygen (balance nitrogen; FiO_2_ 0.10) (BioSpherix, Lacona, NY) as described before^22^. Lung tissues from the PAH mice were stored at −80 °C for subsequent quantitative RT-PCR analyses.

### Vectors

For miRNA overexpression experiments in rat PASMCs, lentiviral vectors based on pLVX-Puro backbone (Clontech, Mountain View, CA) were used to express miR-322 as described before^22^. To study the effect of the miRNAs on HIF-1α accumulation, adenovirus expressing oxygen dependent degradation domains (ODDD: HIF-1αamino acid 531–575) or mutated ODDD (ODDD-mut: P564A) were used to generate cells with or without stable endogenous total α-type HIF proteins as previously reported^22^. Plasmid vectors containing short hairpin RNA targeted to HIF-1α (shHIF-1α), HIF-2α (shHIF-2α) and nonspecific control (shControl) were constructed based on pLVX-hU6. The shRNA sequences containing the *Bam*HI and *Mlu*I restriction sites were synthesized as follows: shHIF-1α, 5- GAT CCG AGA GGT GGA TAT GTC TGG GTC AAG AGC CCA GAC ATA TCC ACC TCT TTT TT -3; shHIF-2α, 5- GAT CCG TTG ATG AAT CCT CGA CTT ATC AAG AGT AAG TCG AGG ATT CAT CAA TTT TT -3; shControl, 5- GAT CCG GGT CTG TAT AGG TGG AGA TCA AGA GTC TCC ACC TAT ACA GAC CCT TTT T -3. Synthetic siRNAs for BMPR1a and Smad5 knockdown were purchased from GenePharma Co.,Ltd (GenePharma, Shanghai, China). Knockdown of miR-322 was achieved by using dTuD constructs against mature rno-miR-322, which was modified from TuD vector^39^ with one more TuD expression cassette. The sequence against cel-miR-39, an elegans miRNA, was used as a negative control. All constructs were subsequently confirmed by DNA sequencing.

### Cells

Rat PASMC cells were isolated from pulmonary arteries of Sprague-Dawley rats. Briefly, segments of the main extra-pulmonary arteries near the hilum were removed under aseptic conditions and dissected free from connective and fat tissues. The medial wall of the pulmonary artery was dissected away from the adventitia and intima and subjected to enzymatic digestion in buffer containing 1 mg/ml collagenase (Worthington Biochemical Corp., Freehold, NJ) and 0.5 mg/ml Elastase type IV (Sigma-Aldrich, St Louis, MO) for 60 min at 37 °C. A7r5, a cell line derived from rat thoracic aorta, and 293a, a cell line derived from human embryonic kidney, were purchased from American Type Culture Collection (Manassas, USA).

### Hypoxia treatment

For hypoxia experiments, cells were placed in a special hypoxia incubator infused with a gas mixture of 5% CO_2_ and nitrogen to obtain 3% oxygen concentration. To chemically stabilize HIFs, cells were treated with 200 μM CoCl_2_ (Sigma-Aldrich) or an equivalent volume of deionized water (dissolvent control) for 24 h.

### Western blotting

Cells were lysed with ice-cold RIPA buffer (50 mM Tris-HCl, pH 7.5; 150 mM NaCl; 1% NP-40; 0.25% sodium deoxycholate, 1mM EDTA), supplemented with a protease inhibitor cocktail (Sigma-Aldrich). Equal amounts of extracts (30μg) were then electrophoresed on a sodium dodecyl sulfate (SDS) polyacrylamide gel and electroblotted to nitrocellulose filter membranes (Millipore). Then, membranes were immersed in blocking buffer (5% degreased milk powder) for 1 h and incubated with antibodies against HIF-1α, HIF-2α (Novus Biologicals, Littleton, CO), Smad5, TATA-binding protein (TBP), α-tubulin, Bmpr2, Bmpr1a (ProteinTech Group, Chicago, IL), β-actin or Smad4 (Santa Cruz Biotechnology, Santa Cruz, CA) overnight at 4°C. They were then incubated with horseradish peroxidase-conjugated secondary antibodies (Jackson Immuno-Research, West Grove, PA) and the protein bands were visualized using the SuperSignal chemiluminescent detection module (Pierce).

### Quantitative RT-PCR

Total RNA was extracted with RNAiso Plus (Takara biotechnology Co., Dalian, China) and quantified using the NanoDrop 2000c Spectrophotometer (Thermo Fisher Scientific, Wilmington, DE). Isolated RNA was polyadenylated with poly(A) Polymerase Tailing Kit (Epicentre) in accordance with the manufacturer’s instructions. Then, reverse transcription and quantitative real-time PCR were performed using the S-Poly (T) method, as previously reported by us^40^. Primers used for reverse transcription and quantitative PCR are as follows: miR-322, RT primer (5- GTG CAG GGT CCG AGG TCA GAG CCA CCT GGG CAA TTT TTT TTT TTT CCA AA -3) and forward primer (5- GCC CGC CAG CAG CAA TTC ATG T -3); sno202 RT primer (5- GTG CAG GGT CCG AGG TCA GAG CCA CCT GGG CAA TTT TTT TTT TTC ATC AG -3) and forward primer (5- GTA CTT TTG AAC CCT TTT CCA T -3).

### Reporter gene assay

HIF-dependent gene regulation was performed by a dual reporter gene assay, using a firefly luciferase construct and a reference Renilla luciferase construct pRL-TK (Promega, Madison, WI). A putative promoter of miR-322 containing the consensus sequence of the HRE was constructed by PCR from rat genomic DNA by using the primers: 5- CCG CTC GAG TAC TTA GTT TAA CTA CAG ACT C -3 (forward) and 5- CGA CGC GTG GGG CCG CTC TGG GGT ACC TGC -3 (reverse). Luciferase activity was measured in cell extracts with a Lumat LB9508 luminometer (Berthold, Bad Wildbad, Germany).

Validation of miR-322 targets was carried out using a 3′ UTR activity assay with a similar firefly/renilla luciferase reporter system (Promega, Madison, WI). The corresponding mutant constructs with five mutated residues in the region of seeding sequence were generated by site-directed mutagenesis. The primers used are as follows: bmpr1a, 5- GTC GAA TTC GAG GGA GAA TTT AGA CTG CAA GAA C -3 (forward) and 5- AGC TCT CGA GCA TAT CAA AGG TAT GGT GCA CCA TC -3 (reverse); smad5, 5- GTC GAA TTC TAC AGA TGC TGT GAG CTG ACA TGG -3 (forward) and 5- AGC TCT CGA GTC TCA TGG AGC TGA CAC ACT CTT GC -3 (reverse); bmpr1a-mut, 5- ACT CCT TCC TCT ACA TCT TCA CAG GGA CGA AAC AGT AAA CCT TAC CGT ACT CTA C -3; smad5-mut, 5- GTG CTC TGT GGC CTT GTT CAG CAT TGT TTC GAC GTT TGG GCC AAC AAT TC -3.

### Chromatin immunoprecipitation (ChIP)

ChIP assays were carried out using the EZ Chip^TM^ Assay kit (Millipore) according to the manufacturer’s instructions. Briefly, cells were crosslinked with 1% formaldehyde at room temperature for 15 min, washed twice in PBS and lysed in SDS lysis buffer. Chromatin fragments were prepared by sonicating lysates on ice. Then, lysates were incubated with antibodies against HIF-1α, HIF-2α (Novus Biologicals), RNA polymerase II, or IgG (Millipore). Immunoprecipitated complexes were collected using protein G-agarose beads. The pellets were washed with Elution buffer and incubated at 65 °C for 4 h to reverse the cross-link by formaldehyde. They were then digested with 50 μg/ml proteinase K for 1 h. Dissociative DNA were purified using the Cycle Pure Kit (Omega) and subjected to PCR amplification. The primers used for amplification of the HRE and negative control segments of the promoter were as follows: HRE, 5 -AGG AAA TGA ATC AAT GTA AC -3 (forward) and 5- TAA GCA ACT CCA CCA AAA -3 (reverse); negative control, 5- TCC GCT CTT CTT CCC TCA -3 (forward) and 5– TGC GAA GCT GCT CAG TCG -3 (reverse).

### Cell cycle analysis

Approximately 1 × 10^6^ cells were treated with serum-depleted medium (0.1% FBS) for 24 h to synchronize growth. Then cells were incubated in complete culture medium for another 24 h, detached by trypsinization, stored in culture medium and recovered by centrifugation. After fixation with 75% ethanol overnight at 4 °C, cells were digested with DNase-free RNase in PBS containing 5 μg/ml propidium iodide (PI) for DNA staining (30 min at 37 °C). FACS analysis was performed using a BD FACS Calibur flow cytometer equipped with CELL Quest software.

### Immunocytochemistry

For immunocytochemical analysis, cells were fixed in 4% paraformaldehyde for 20 min, and permeabilized with 0.5% Triton X-100 for 20 min. Pretreated cells were incubated with a primary antibody against smooth muscle α-actin (α-SMA) (Sigma-Aldrich) diluted 1:200. Indirect immunofluorescence was observed after incubation with a secondary Alexa 488-conjugated anti-mouse IgG (Jackson Immuno-Research) diluted 1:500. Nucleic acid dye 4′, 6′-diamidino-2-phenylindole (DAPI) staining was performed to detect nuclei.

### Measurement of cell proliferation and migration

Cell proliferation was determined by cell viability assay and EdU incorporation assay. Approximately 4 × 10^3^ cells were seeded in triplicate in 96-well plates. Cell viability was assessed using the CellTiter 96 Aqueous One Solution Proliferation Assay Kit (Promega). EdU labeling was performed using the EdU Assay Kit (Ribobio, Guangzhou, China) as recommended by the manufacturer. Cell migration was determined by wound-healing assay as previously described^7^ by measuring the decreased width of the scratch.

### Statistical analysis

All data shown are mean values of at least three experiments, each performed in triplicate, with standard deviation (SD). When only two groups were compared, the statistical differences were assessed with the double-sided Student’s *t* test. Significant differences between groups were analyzed using one-way ANOVA. A *p* value less than 0.05 was considered significant.

## Additional Information

**How to cite this article**: Zeng, Y. *et al.* Hypoxia inducible factor-1 mediates expression of miR-322: potential role in proliferation and migration of pulmonary arterial smooth muscle cells. *Sci. Rep.* **5**, 12098; doi: 10.1038/srep12098 (2015).

## Supplementary Material

Supplementary Information
