# Supplementary material for: Hypoxia inducible factor-1 mediates expression of miR-322: potential role in proliferation and migration of pulmonary arterial smooth muscle cells
Source: Sci Rep. 2015 Jul 13;5:12098. doi: 10.1038/srep12098 (PMC4499844; doi:10.1038/srep12098)
Supplement: Supplementary Information [file srep12098-s1.pdf]

Supplementary information

Hypoxia inducible factor-1 mediates expression of miR-322: potential role in proliferation and migration of pulmonary arterial smooth muscle cells

Yan Zeng[1,2*], Hongtao Liu[1*], Kang Kang[1], Zhiwei Wang[3], Gang Hui[4], Xiaoying Zhang[1], Jiasheng Zhong[1], Wenda Peng[2], Ramaswamy Ramchandran[5], J. Usha Raj[5], Deming Gou[1,5#]

[1] Shenzhen Key Laboratory of Microbial Genetic Engineering, College of Life Sciences, Shenzhen University, Shenzhen, Guangdong, 518060, China;

[2] Key Laboratory of Optoelectronic Devices and Systems of Ministry of Education and Guangdong Province, College of Optoelectronic Engineering, Shenzhen University, Shenzhen, Guangdong, 518060, China;

[3] Department of Cardiovascular Surgery, Shenzhen Sun Yat-Sen Cardiovascular Hospital, Shenzhen, Guangdong, 518000, China;

[4] Department of Chest Surgery, Peking University Shenzhen Hospital, Shenzhen, Guangdong, 518000, China;

[5] Department of Pediatrics, University of Illinois at Chicago, Chicago, IL 60612, U.S.A.

*These authors contributed equally to this work.

[#]**Corresponding Author**

Address correspondence to: Deming Gou, PhD, College of Life Sciences, Shenzhen University, Nanhai Ave 3688, Shenzhen, Guangdong, 518060, China. Tel: 86-755-26527848; Fax: 86-755-26534274; E-mail: dmgou@szu.edu.cn

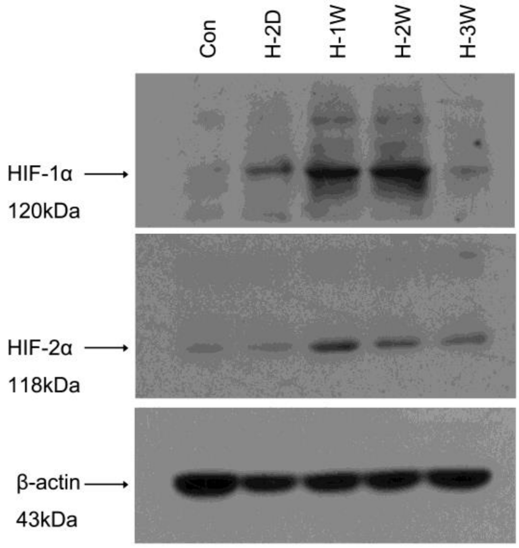

Figure S1. Full-length blots of HIF-1α, HIF-2α and β-actin.

Regions of interest are highlighted in Fig. 1c.

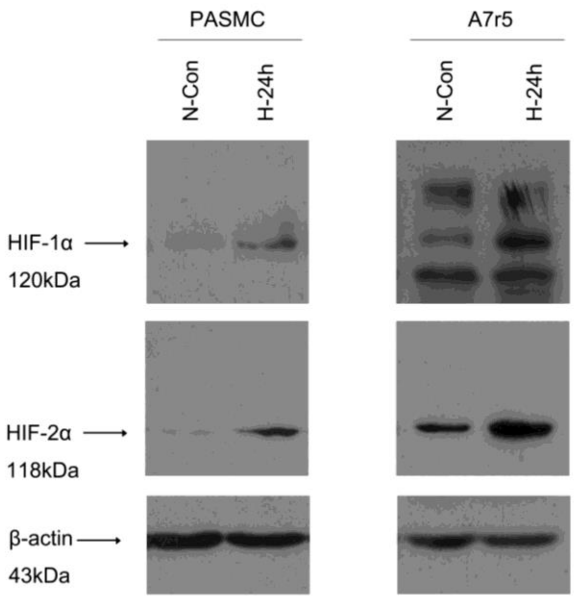

Figure S2. Full-length blots of HIF-1α, HIF-2α and β-actin.

Regions of interest are highlighted in Fig. 2d.

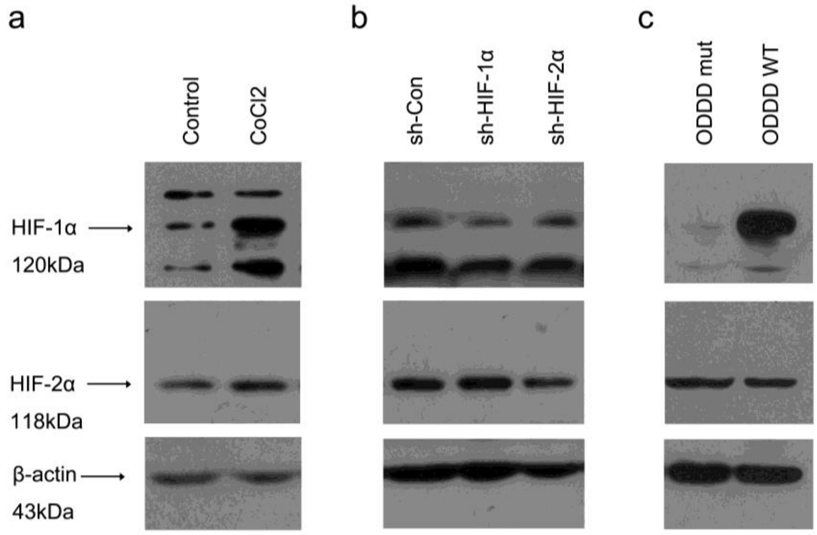

Figure S3. Full-length blots of HIF-1α, HIF-2α and β-actin.

Regions of interest are highlighted in Fig. 3b, 3c and 3f.

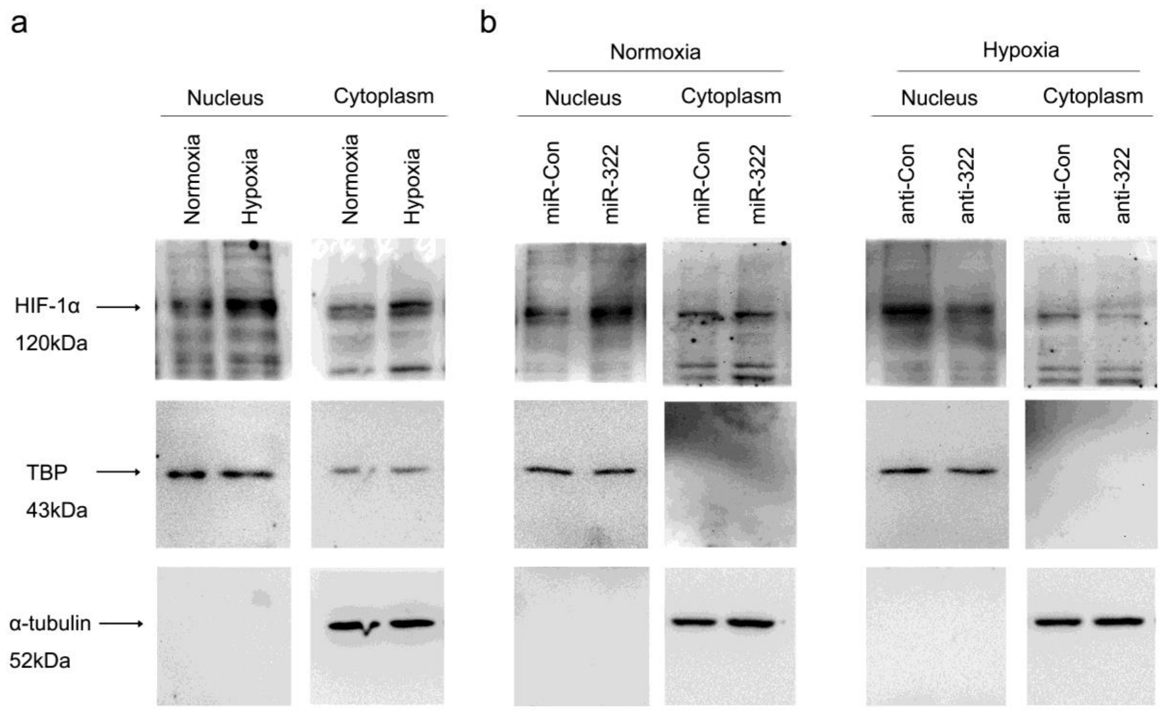

Figure S4. Full-length blots of HIF-1α, TBP and α-tubulin.

Regions of interest are highlighted in Fig. 4a and 4c.

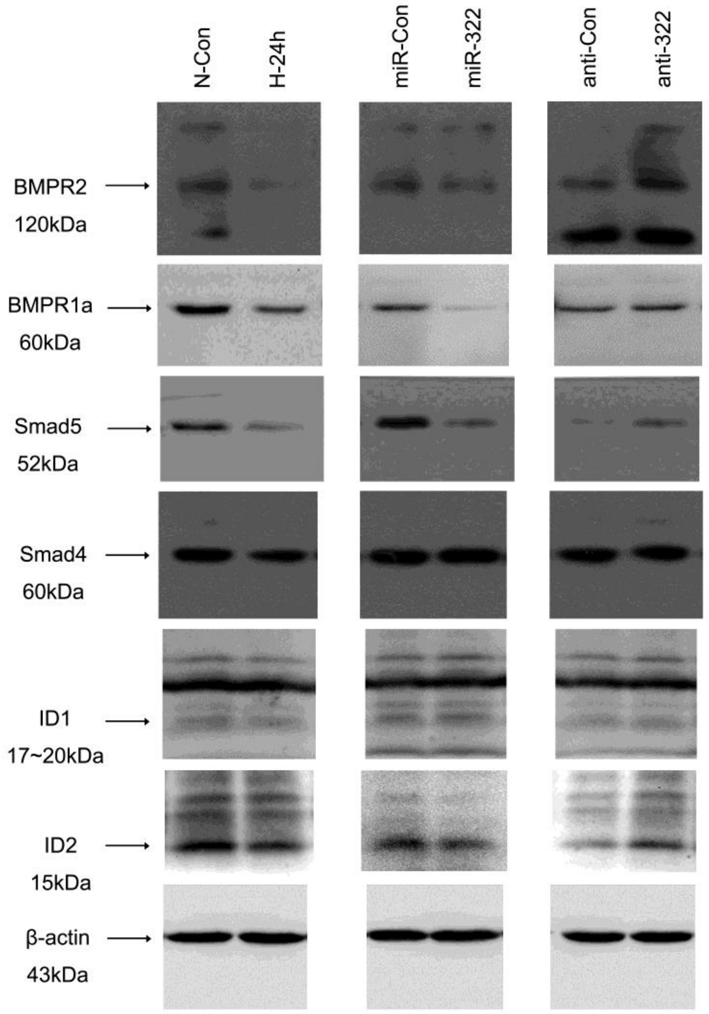

Figure S5. Full-length blots of BMP-Smad signaling pathway.

Regions of interest are highlighted in Fig. 7a.
